# Low flow anesthesia: Efficacy and outcome of laryngeal mask airway versus pressure–optimized cuffed–endotracheal tube

**DOI:** 10.4103/1658-354X.62607

**Published:** 2010

**Authors:** Zeinab A El-Seify, Ahmed Metwally Khattab, Ashraf Shaaban, Dobrila Radojevic, Ivanka Jankovic

**Affiliations:** Department of Anesthesia, Doha Clinic Hospital, Doha, Qatar

**Keywords:** *Endotracheal intubation*, *laryngeal mask airway*, *low flow anesthesia*

## Abstract

**Background:**

Low flow anesthesia can lead to reduction of anesthetic gas and vapor consumption. Laryngeal mask airway (LMA) has proved to be an effective and safe airway device. The aim of this study is to assess the feasibility of laryngeal mask airway during controlled ventilation using low fresh gas flow (1.0 L/min) as compared to endotracheal tube (ETT).

**Patients and Methods:**

Fifty nine non-smoking adult patients; ASA I or II, being scheduled for elective surgical procedures, with an expected duration of anesthesia 60 minutes or more, were randomly allocated into two groups - Group I (29 patients) had been ventilated using LMA size 4 for females and 5 for males respectively; and Group II (30 patients) were intubated using ETT. After 10 minutes of high fresh gas flow, the flow was reduced to 1 L/min. Patients were monitored for airway leakage, end-tidal CO_2_(ETCO_2_), inspiratory and expiratory isoflurane and nitrous oxide fraction concentrations, and postoperative airway-related complications.

**Results:**

Two patients in the LMA-group developed initial airway leakage (6.9%) versus no patient in ETT-group. Cough and sore throat were significantly higher in ETT patients. There were no evidences of differences between both groups regarding ETCO_2_, uptake of gases, nor difficulty in swallowing.

**Conclusion:**

The laryngeal mask airway proved to be effective and safe in establishing an airtight seal during controlled ventilation under low fresh gas flow of 1 L/min, inducing less coughing and sore throat during the immediate postoperative period than did the ETT, with continuous measurement and readjustment of the tube cuff pressure.

## INTRODUCTION

The use of low flow anesthetic techniques with circle absorber breathing systems has become almost the role in modern anesthetic practice, aiming to minimize waste of expensive volatile anesthetics, reduce atmospheric pollution, and moreover conserving airway humidity and body temperature.[1]

Baker in 1994 had suggested the following classification of flow rates of gases into anesthetic circuits: Minimal flow = 0.5 L or less fresh gas flow (FGF) per min.

Low flow = ≥ 0.5 - 1 L/min.

Medium flow = ≥ 2 - 4 L/min.

Very high flow= ≥ 4 L/min.[2]

An airtight seal between the airway device and patient airway is essential to establish a leak-free semi-closed circuit system, particularly during controlled lung ventilation.[3] The laryngeal mask airway (LMA) has proved to be an effective and safe airway device. However, there were controversies over its ability deliver positive pressure ventilation, particularly under reduced gas flow rates and for prolonged procedures.[4]

Postoperative airway complications such as sore throat, cough and difficulty in swallowing are common complaints following either endotracheal tube (ETT) or LMA. Meta-analysis of studies comparing the LMA-classic and laryngoscope-guided tracheal intubation revealed that the incidence of sore throat is much higher for tracheal intubation.[5] Cuff pressure greater than 30 cm H_2_O, the use of nitrous oxide, and the lack of frequent Adjustment of cuff pressure is thought to be the most important factor that contributes to postoperative airway complications even in minor procedures.[6,7]

The present study aims to test the feasibility of using LMA (size 4 for females and 5 for males) during controlled positive pressure ventilation in adult patients under low fresh gas flow of 1 L/min.

## PATIENTS AND METHODS

This is a prospective, randomized, patient-blind, single-center study carried out after approval of the hospital medical Ethics Committee. Fifty-nine, non-smoking, ASA I and II patients, aged between 19 to 50 years old were scheduled for elective surgical procedures during general anesthesia with an expected duration of anesthesia 60 minutes or more, (operations lasted less than 45 minutes, have been excluded). Patients were randomly enrolled to participate in this study, after taking informed patient consent. A power analysis was performed to determine the number of patients needed to detect a 50% difference in the leak fraction between the two airway devices based on the previously published variability of the leak fraction when an LMA device (n = 24) is used. A mean leak fraction of 0.25 with a standard deviation of 0.15, an alpha error of 0.05, and a power of 80% were used in the calculation.[8] Because we expected some exclusions and failure to follow-up during the course of the study, we increased the number of the sample size of each group to (n = 31) as one with unexpected difficult airway and two patients scheduled for LMA insertion but failed due to abnormal anatomy and were intubated where excluded.

Exclusion criteria were: expected difficult airway, recent history of sore throat or common cold within the last 10 days, patients with known allergy to latex, patients with full stomach. Patients were premedicated with oral midazolam 7.5 mg 1 hour before surgery. In all patients, anesthetic induction was performed with propofol (Diprivan^®^1% Astra-Zeneca, Madrid) 2-3mg/kg, fentanyl 2μg/kg, cisatracurium (Nimbex^®^ -GlaxoSmithKline, S.A. Spain) 0.15mg/kg. Mask ventilation with 100% oxygen (6 Ls/min), and isuflorane 2 ± 0.5 Vol% started for 2.5 -3 minutes. Intubation or laryngeal mask insertion was achieved after ensuring suitable conditions. Lubricated, sterile ETTs (RÜSCH), manufactured by RÜSCH Uruguay Ltd. of ID 7.5 (for women) and 8 (for men) and laryngeal mask airways (LMA-Classic™) of size 4 (for women) and size 5(for men) were used. Cuff inflation of ETT was adjusted to be close to 25 cm H_2_ O using Endotest (RÜSCH) that was connected to the tube for continuous pressure readjustment (the cuff pressure changed and readjusted during surgery but we did not record its incidence).While LMA was inflated until "just seal" obtained, adequacy of seal was decided on the basis of auscultation of anterior neck and ballottement of pilot balloon[9] (the position of the LMA was verified clinically only and we depend on disappearance of leakage during its inflation but we did not measure its cuff pressure).

Lungs were mechanically ventilated with 35:65% oxygen and nitrous oxide. Maintenance of anesthesia was achieved with isoflurane (Abbott) 2.0 ± 0.50 Vol % in fresh gas flow of (6 Ls/min) for 10 minutes to deliver sufficient isoflurane and N_2_O during the high-uptake period. Flows were, then reduced to 1 L/min; isoflurane vapor setting to 1.0 ± 0.5 Vol %. In case of insufficient anesthesia, fentanyl (50-100 μg) was given. German Dräger (Julian) anesthesia machines were used, and the patients' were ventilated mechanically at respiratory rate 11 per min, tidal volume (ml) = weight (Kg) × 8 ml, positive end expiratory pressure (PEEP) 3 cm water, The anesthesia delivery system (Dräger Medizintechnik GmbH 23542 Lübeck, Germany) was modified with soda–lime CO_2_ absorber. All patients were monitored using electrocardiography, pulse oximetry, non-invasive blood pressure, inspiratory and expiratory N_2_O, and isoflurane concentrations; and the peak and plateau ventilatory pressure, End-tidal CO_2_ analysis were also monitored continuously by the anesthesia machine but only recorded: after induction, 10, 20, 40, 60, 90, and 110 minutes later. The occurrence of rebreathing (inspiratory CO_2_ > 5 mmHg) and system leakage were monitored by the anesthesia machine (system leakage must not exceed 100 ml/min). Any attempts to increase fresh gas flow secondary to leakage (when the ventilator bellows fail to rise to the top of its clear plastic enclosure during expiration) were noticed and recorded. A short period of high fresh gas flow (2.5 Ls/min) was then applied, to overcome this problem. If correctable, the fresh gas flow was reduced again to 1 L/min.

Isoflurane was discontinued eight minutes before the end of surgery and the flow increased to 6 Ls/min 100% O_2_to wash-out anesthetics. After removal of the airway device, patients were transferred to the post-anesthesia care unit (PACU). The incidence and severity of cough, sore throat, and difficulty in swallowing were assessed separately one hour after removal of airway device using visual analogue scale (VAS) {0 = no complaint → 10 = worst imaginable complaint}

### Statistical methodology

Analysis of data was done by IBM computer using SPSS (statistical program for social science) as follows:

- Description of quantitative variables as mean, SD and range
- Description of qualitative variables as number and percentage.
- Chi-square test was used to compare qualitative variables
- Unpaired *t*-test was used to compare a quantitative variable between two independent groups in parametric data.
- *P* value > 0.05 insignificant
- *P* < 0.05 significant
- *P* < 0.01 highly significant

## RESULTS

Fifty-nine patients (30 of them were managed using ETT and 29 with LMA) were included in this study. Another three patients were excluded from the study, one with unexpected difficult airway and two patients scheduled for LMA insertion but failed due to abnormal anatomy and were intubated. Demographic data, duration of anesthesia, duration of airway devices, intraoperative fentanyl consumption, and duration of stay in PACU are presented in Table 1, and showed no significant differences.

**Table 1:** Patients demographic and operative data

	**LMA**	**ETT**
No	29	30
Age (year)	39.06 ± 5.5	38.2 ± 5.8
Sex (M/F)	25/8	21/9
Duration of anesthesia (min)	81.13 ± 16.21	87.8 ± 15.33
Duration of airway device (min)	91.82 ± 16.66	98.53 ± 16.64
Fentanyl consumption (μg)	183.44 ± 36.97	194.6 ± 39.4
Stay in (PACU) (min)	17.93 ± 1.85	19.73 ± 2.17
*t* = 0.7	*p* 0.05	χ^2^ = 0.97

* Significant test P < 0.05

** Highly significant test P < 0.01

**Figure 1:**
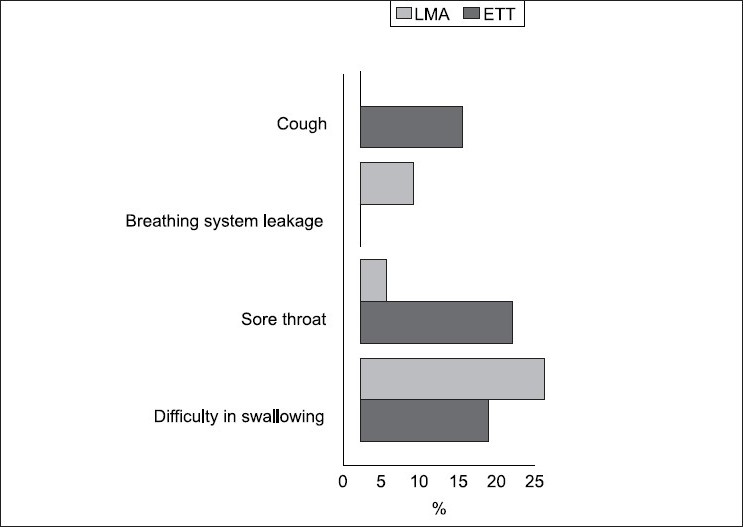
Postoperative airway-related complications and breathing system leakage

System leakage was noticed in two patients of LMA-group (6.9%), which was corrected by increasing the flow to 2.5 Ls/min for a short period to refill the ventilator bellows and this was repeated twice in one patient, the flow was then reduced to 1 L/min and continued to the end of operation, Statistically there was no significant difference between both groups regarding system leakage (*P* > 0.05) [Figure 1].

**Figure 2:**
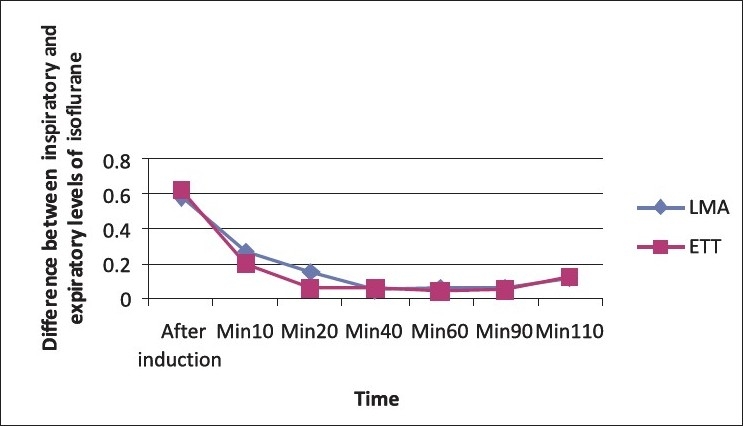
Comparison between LMA and ETT regarding uptake of isoflurane through the operation

There was no recorded re-breathing in all patients, inspiratory CO_2_did not exceed 4 mmgh in any patient, and ET-CO_2_was within normal ranges in both groups, with no significant difference between both groups regarding ETCO_2_ or uptake of gases during the operation [Figure 2].

One hour after removal of airway device, four patients in ETT-group (13.3%) reported mild cough with VAS (0.4 ± 1.06), while six patients (20%) of the same group complained of sore throat with VAS (0.83 ± 1.83) versus non of patients and one patient (3.4%) with VAS (0.068 ± 0.37) respectively in LMA group (*P* < 0.05), meanwhile there were no significant difference in the incidence or severity of the difficulty in swallowing between both groups (*P* > 0.0.5), as shown in and Figure 1.

There was no recorded obligation to increase inspiratory isoflurane concentration above the pre-determined range to compensate for any leakage.

There was no recorded hypoxia (SPO_2_ < 94%), or gastric distension in all patients under the current study.

## DISCUSSION

The current study has been designed to test the efficacy and safety of LMA in replacing ETT during relatively – lengthy operations under controlled ventilation using 1L/min fresh gas flow. The net result of our study showed that LMA was as effective and safe as ETT in majority of patients enrolled in the study (only 6.9 % of LMA patients developed initial airway leakage that has been shown to be correctable by transient increase in fresh gas flow). The absence of significant CO_2_- rebreathing in all LMA-patients and insignificant difference in anesthetics uptake between both groups re-inforced this suggestion.

The LMA is an established method of airway control during spontaneous ventilation.[10–12] On the other hand, some earlier studies[13,14] failed to confirm this proposal during controlled mechanical ventilation, until a mass survey of 11,910 patients had been published in 1996[15] and demonstrated the efficacy and safety of LMA during controlled mechanical ventilation, mentioning that the earlier controversies may be related to the lack of experience of proper usage and insertion.

Few studies have tested the feasibility of the LMA under low flow conditions. Möllhoff *et al*. in 1996[16] used LMA during low and minimal –flow anesthesia in patients undergoing short elective gynecological surgery. There was no ETT- control group in this study. Similarly, Honemann and his colleagues in 2001[17] compared LMA to ETT under minimal flow conditions in relatively lengthy operations. The results of both studies were supporting the concepts of efficacy and safety of LMA. In the later study significant initial leak were reported in 15.4 % of LMA –patients in comparison to only 6.9 % reported in our study.

There are accumulating evidences supporting the use of LMA size 4 for females and size 5 for males. AS a sex-related formula is a more successful strategy than the manufacturer′s weight-based recommendations in avoiding air leak from the gap between the mask and pharynx[9,18,19] This also was found to decrease the incidence of postoperative airway complications resulting from pharyngeal wall ischemia and lingual nerve injury due to over inflation of small size LMA.[20]

In the present study, in spite of continuous optimization of ETT-cuff pressure to be around 25 cm H_2_O, the incidence of cough and sore throat in intubated patients were shown to be significantly higher than those occurring with LMA, 13.3% of ETT-patients complained of cough and 20% of them complained of sore throat in comparison to 0% and 3.4% in LMA-patients respectively. The VAS in patients reporting sore throat showed a greater degree in ETT-patients. On the other hand, there were minimal differences between both groups regarding swallowing problems. One study failed to show any protective effect of limiting ETT-cuff pressure on post-intubation sore throat incidence.[21] Another recent study mentions that mechanical irritation and stretch caused by ETT cuff, even in low pressure conditions, can stimulate the sensory C and afferent delta fibers present in the larynx and trachea and eliciting postoperative airway symptoms.[22] On the other hand, there was no evidence that LMA–cuff pressure monitoring and limitation is necessary during LMA anesthesia. Expansion of LMA –cuff secondary to diffusion of N_2_O does not cause displacement of the cuff from the hypo- pharynx and is self-limiting.[23]

In conclusion, the laryngeal mask airway has been proved to be effective and safe in establishing an airtight seal during controlled ventilation under low fresh gas flow of one L/min. The laryngeal mask airway induced less coughing and sore throat during the immediate postoperative period than did the ETT in-spite of optimization of its cuff pressure.
